# The Green Synthesis of 2D Copper Nanosheets and Their Light Absorption

**DOI:** 10.3390/ma14081926

**Published:** 2021-04-12

**Authors:** Suhyun Lee, Suming Wang, Chien Wern, Sung Yi

**Affiliations:** Department of Mechanical and Materials Engineering, Portland State University, Portland, OR 97207-075, USA; suhyun@pdx.edu (S.L.); wangsuming1993@gmail.com (S.W.); wernc@pdx.edu (C.W.)

**Keywords:** synthesis, 2D nanomaterial, nanosheets, 2D copper nanosheets

## Abstract

In this study, a new green synthesis method for two-dimensional (2D) copper nanosheets is developed using methylsulfonylmethane (DMSO_2_). The chemical composition and light absorption of 2D copper nanosheets are also studied. A new green method is mainly to utilize DMSO_2_, which is environmentally friendly enough to be considered a food-grade chemical, unlike the conventional method using toxic chemicals, such as ammonia and hydrazine (N_2_H_4_). With a reducing agent, the aggregation of uncertain copper products was produced in the absence of DMSO_2_, while 2D copper nanosheets were formed in the presence of DMSO_2_. The optimum concentration of DMSO_2_ as a surfactant was determined to be 2 M, resulting in large surface areas with regular edges. FTIR spectrum confirmed C–H bonding from DMSO_2_ used to synthesize 2D copper nanosheets. The light absorption peak was revealed at 800 nm in the UV–vis spectrum. This proposed new green method not only has a simpler process than the conventional methods, such as hydrothermal method and chemical bath deposition, but also substitutes toxic chemicals with DMSO_2_. 2D copper nanosheets can be used for various applications, including conductive filler or ink in the flexible electronics and laser photonics fields.

## 1. Introduction

Metal nanomaterials have attracted interest due to their great transmittance and electrical, thermal, mechanical, and catalyst properties. They enable extensive applications in nanoscience and nanotechnology, such as flexible electronics [1]. In particular, two-dimensional (2D) nanomaterials have unconventional exposed surface properties indicating high electron mobility and good thermal conductivity [2,3,4,5]. Besides, 2D nanomaterials can provide ideal morphology for vigorous electrocatalysts because the pathways of ion and electron diffusion are significantly shortened, and electrochemically active sites are large [6,7].

2D nanosheets are a promising nanomaterial due to their thickness of nanometers along with in-plane lattice and pores between interlayers. 2D nanosheets offer minimized transport resistance and maximized selectivity depending on molecular size [8]. Single-layer manganese dioxide (MnO_2_) nanosheets and ZnMn_2_O_4_–graphene (ZMO-G) nanosheets demonstrated attractive electrochemical properties, such as high capacitance, and better rate performance for electrical energy storage system [9,10,11]. Furthermore, graphene and graphene oxide nanosheets were investigated in energy-related areas, water desalination, and filtration as functional separation membranes [8,12]. 2D copper oxide (CuO) nanosheet electrode film was found to be a low-cost catalyst material with great catalytic activity for electrochemical water splitting into hydrogen and oxygen [6]. Copper (Cu) nanosheet ink presented stable conductive performance and could be used directly on flexible electronic circuits [13].

The synthesis methods of 2D metal nanomaterials are largely categorized into two methods, namely top-down and bottom-up methods. Top-down methods are mainly employed for producing layered bulk material, while bottom-up methods are widely utilized in practical applications [14]. 2D metal nanosheets are dominatingly synthesized through bottom-up methods due to the advantage of practical application [15]. Among bottom-up methods, hydrothermal and solvothermal methods are used to synthesize metal 2D nanosheets.

Dang et al. (2014) and Dehghanpour et al. (2015) employed the hydrothermal method to produce Cu nanosheets using polyvinylpyrrolidone (PVP) and cetyltrimethylammonium bromide (CTAB), respectively, as surface capping agent [13,16]. Hou et al. (2013) and Duan et al. (2014) presented the synthesis of rhodium (Rh) nanosheets by the solvothermal method in the presence of surfactants and the absence of surfactants, respectively [17,18]. Besides these methods, Hu et al. (2010) prepared metal oxide nanosheets using several metals (e.g., Mn, Co, Fe, and Ni) by refluxing metal salt in absence of any surfactants, or templates [19]. Dubal et al. (2010) and Pawar et al. (2017) synthesized CuO nanosheets using ammonia as a reducing agent through chemical bath deposition [6,20]. Shaik et al. (2016) demonstrated the synthesis of Cu nanosheets through the reduction of copper salts at room temperature in the presence of CTAB as a surfactant and hydrazine (N_2_H_4_) as a reducing agent [21]. The foregoing methods require complicated processes, high temperature, and prolonged reaction time. 

In recent years, significant attention has been given to various 2D metal nanomaterials such as manganese (Mn), cobalt (Co), nickel (Ni), and rhodium (Rh) [17,22,23,24]. However, one of the important nanomaterials is copper due to its high conductivity, low cost, high abundance, and even flexibility in the form of nanowires. Furthermore, 2D copper nanosheets demonstrated higher conductivity and lower resistivity than copper nanowires or mixed products of copper nanowires and 2D nanosheets [25]. Copper-based nanomaterials have been presented in many applications such as electrocatalysts for water oxidation [26], photodetectors [27], conductive ink [13], supercapacitors [20], and conductive fillers [25]. Therefore, 2D copper nanosheets are considered promising nanomaterials for the substitution of novel metal nanomaterials. However, the sources for the synthesis of 2D copper nanosheets are scarce. In addition, the synthesis methods of 2D copper nanosheets require toxic chemicals, such as ammonia and N_2_H_4_, and are implemented with complicated procedures [6,20,21].

In this present study, a new green method to synthesize 2D copper nanosheets is developed using methylsulfonylmethane (DMSO_2_), which has not been reported before for the synthesis of nanomaterials. DMSO_2_ is environmentally friendly enough to be used as a food-grade chemical and can be used to substitute the toxic chemicals, such as ammonia and N_2_H_4_, used in the conventional method. In addition, two reducing agents, namely sodium hydroxide (NaOH) and lithium hydroxide (LiOH), are employed. 2D copper nanosheets are characterized by transmission electron microscopy (TEM), energy-dispersive X-ray spectroscopy (EDX), Fourier transform infrared spectroscopy (FTIR), and ultraviolet–visible spectroscopy (UV–Vis).

## 2. Materials and Methods

### 2.1. Materials

For the synthesis of 2D copper nanosheets, copper sulfate (CuSO_4_) was utilized as the copper precursor. Sodium hydroxide (NaOH) and lithium hydroxide (LiOH) were used as reducing agents to reduce copper ions in the solution. Methylsulfonylmethane (DMSO_2_, >99.8%, Bergstrom Nutrition, Vancouver, WA, USA) was employed to determine whether it plays the role of a surfactant in the synthesis process. Deionized water (DI H_2_O) was used to dissolve all the chemicals.

### 2.2. Method

2D copper nanosheets were synthesized by a new green method. In order to keep constant reaction temperature for 2 h reaction time, the synthesis was carried out by placing an Erlenmeyer flask containing material solution into the water bath. In addition, all steps were implemented under magnetic stirring at 300 rpm to keep the solution in a homogeneous state. First, 1, 2, or 3 M of methylsulfonylmethane (DMSO_2_), 0.01 M of copper sulfate (CuSO_4_), and 0.3 M of reducing agents were dissolved with deionized water (DI H_2_O) in each Erlenmeyer flask, separately. CuSO_4_ solution was added to DMSO_2_ solution (1, 2, or 3 M) under magnetic stirring for 10 min, and then 0.05 mL of 0.3 M sodium hydroxide (NaOH) reducing agent was added. This solution in an Erlenmeyer flask was stirred for 30 min at a water-bath temperature of 80 ℃. When using a lithium hydroxide (LiOH) reducing agent instead of NaOH, the process of synthesis and concentration of materials was kept the same. After 30 min, the solution was washed with methanol to remove impurities of chemicals and dried in a vacuum desiccator at room temperature under full vacuum for at least 2 h. 

### 2.3. Characterization

The morphology and arrangement of 2D copper nanosheets were investigated by transmission electron microscopy (TEM, Tecnai F-20, FEI, Hillsboro, OR, USA) with 200 kV accelerating voltage, and energy-dispersive X-ray spectroscopy (EDX) was employed for elemental analysis of 2D copper nanosheets. To analyze the composition of 2D copper nanosheets, Fourier transform infrared spectroscopy (FTIR, Thermo Fisher, Hillsboro, OR, USA) was used. The light absorption spectrum of 2D copper nanosheets was obtained by ultraviolet–visible spectroscopy (UV–Vis, Evolution 260 BIO, Thermo Fisher, Hillsboro, OR, USA).

## 3. Results and Discussion

### 3.1. The Formation of 2D Copper Nanosheets

**Figure 1:**
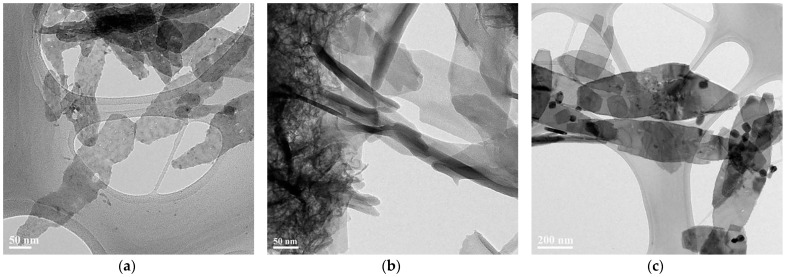
TEM images of 2D copper nanosheets: (**a**) without NaOH reducing agent; (**b**) without DMSO_2_; (**c**) with NaOH reducing agent and DMSO_2_.

2D copper nanosheets were synthesized by adding methylsulfonylmethane (DMSO_2_), copper sulfate (CuSO_4_), and sodium hydroxide (NaOH) together. As shown in Figure 1, 2D copper nanosheets were formed under various conditions. Figure 1a,b show 2D copper nanosheets synthesized without NaOH reducing agent and DMSO_2_, respectively. Figure 1c represents 2D copper nanosheets synthesized with NaOH reducing agent and DMSO_2_. The chemical reaction can be represented as follows:

(1) $${\mathrm{CuSO}}_{4}\;+\;2\mathrm{NaOH}\;\to \mathrm{Cu}(\mathrm{OH}{)}_{2}\;+\;{\mathrm{Na}}_{2}{\mathrm{SO}}_{4}$$

(2) $$\mathrm{Cu}(\mathrm{OH}{)}_{2}\;+\;\mathrm{NaOH}\;\to {\mathrm{Na}}_{2}{{\mathrm{CuO}}_{2}}^{-}\;+\;H_{2}O\;+\;H^{+}$$

(3) $${\mathrm{Na}}_{2}{{\mathrm{CuO}}_{2}}^{-}\;+\;{H^{+}}_{\;}\to \mathrm{Cu}\;+\;2\mathrm{NaOH}$$

The surfaces of 2D copper nanosheets were 0.365 µm in length and 0.1 µm in width in the absence of a NaOH reducing agent, as shown in Figure 1a. These 2D copper nanosheets display irregular edges and some pores on the surface. This may result from an incomplete reduction process of copper ions due to the fast oxidation of 2D copper nanosheets. The 2D copper nanosheets produced without DMSO_2_ are shown in Figure 1b. A complete reduction process seems to have occurred, as the edges are regular and improved when compared to those shown in Figure 1a. However, the remarkable aggregation of the uncertain copper products makes it difficult to measure the length and width of these 2D copper nanosheets. This aggregation could be caused by the lack of surface energy due to the absence of surfactant in the solution. As shown in Figure 1c, 2D copper nanosheets synthesized with NaOH reducing agent and DMSO_2_ were 0.88 µm in length and 0.32 µm in width, revealing regular edges and larger surface areas when compared to those shown in Figure 1a. In addition, there is no aggregation of the uncertain copper products, unlike that seen in Figure 1b, even though some copper nanoparticles were formed simultaneously. This may be caused by a fast reduction reaction and nucleation rate because NaOH is a strong reducing agent. 

DMSO_2_ may play a role as a copper surfactant in this process and be an important factor in forming 2D copper nanosheets of large surface areas with regular edges. This is because copper ions cannot have a driving force to grow desirable 2D nanosheets without copper surfactant. To synthesize 2D copper nanosheets of large surface areas with regular edges, the concentration of DMSO_2_ was controlled.

**Figure 2:**
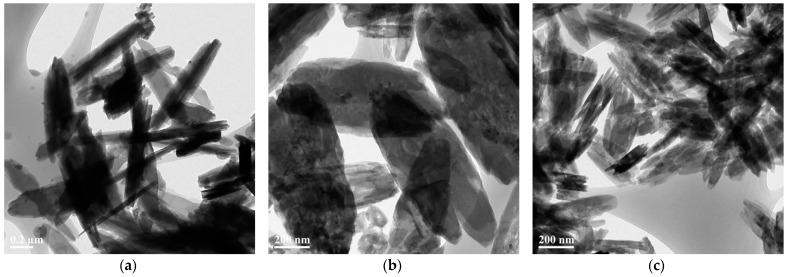
TEM images of 2D copper nanosheets: (**a**) 1 M; (**b**) 2 M; (**c**) 3 M of DMSO_2_.

Figure 2 presents the 2D copper nanosheets synthesized with various concentrations of DMSO_2_. The 2D copper nanosheets synthesized with 1 M of DMSO_2_ shown in Figure 2a reveal a length of 0.95 µm and a width of 0.2 µm. These 2D copper nanosheets were irregularly synthesized, as indicated by the small surface areas and the aggregation of 2D copper nanosheets shown in Figure 2a. Besides, copper products in the form of nanorods and needles can be observed. When 2 M of DMSO_2_ was added, 2D copper nanosheets 0.955 µm in length and 0.42 µm in width were found to have large surface areas with regular surface edges, as shown in Figure 2b. With further increase in the concentration of DMSO_2_, the yield of 2D copper nanosheets began to develop as shown in Figure 2c. However, these 2D copper nanosheets seem to be 0.4 µm in length and less than 0.2 µm in width with surface areas that are not as large as those of nanosheets synthesized with 2 M of DMSO_2_. The irregular edges and aggregation of 2D copper nanosheets can be observed. Furthermore, Figure 2c shows that 2D copper nanosheets seem to form multiple layers, making it difficult for them to disperse. 

The low concentration of DMSO_2_ is likely to cause high surface tension and low surface energy. This makes it difficult for the copper ions to form large areas. On the other hand, the high concentration of DMSO_2_ may cause low surface tension and high surface energy, making the copper ions actively react with each other. However, the excess amount of DMSO_2_ is likely to lead to fast crystallization that limits the growth of 2D copper nanosheets. Therefore, DMSO_2_ added at a sufficient concentration (i.e., 2 M of DMSO_2_ in this study) could act as a surfactant in the synthesis process. DMSO_2_ is environmentally friendly enough to be used as a food-grade chemical. Thus, DMSO_2_ may be used as a substitute for the toxic chemicals, such as ammonia and N_2_H_4_, used in the conventional method.

Lithium hydroxide (LiOH) is used as a weak reducing agent to prevent the generation of by-products of copper nanoparticles. The chemical reaction can be represented by the same mechanism as chemical reactions (1)–(3):

(4) $${\mathrm{CuSO}}_{4}\;+\;2\mathrm{LiOH}\;\to \mathrm{Cu}(\mathrm{OH}{)}_{2}\;+\;{\mathrm{Li}}_{2}{\mathrm{SO}}_{4}$$

(5) $$\mathrm{Cu}(\mathrm{OH})2_{\;}+\;\mathrm{LiOH}\;\to {\mathrm{Li}}_{2}{{\mathrm{CuO}}_{2}}^{-}\;+\;H_{2}O\;+\;H^{+}$$

(6) $${\mathrm{Li}}_{2}{{\mathrm{CuO}}_{2}}^{-}\;+\;{H^{+}}_{\;}\to \mathrm{Cu}\;+\;2\mathrm{LiOH}$$

**Figure 3:**
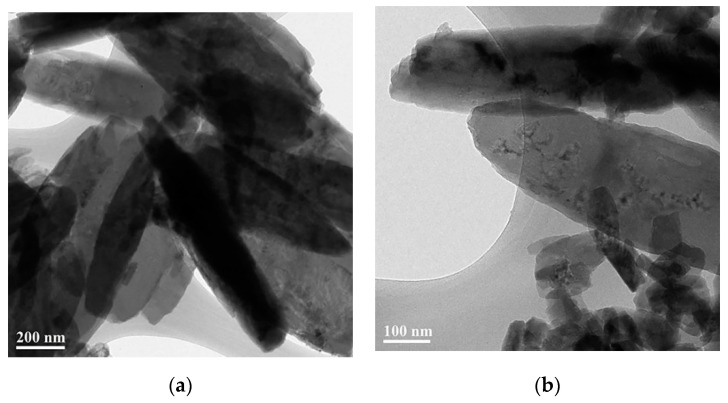
TEM images of 2D copper nanosheets synthesized with the LiOH reducing agent: the magnification of (**a**) 200 nm; (**b**) 100 nm.

Figure 3 shows the morphology of 2D copper nanosheets synthesized with LiOH. These 2D copper nanosheets had a length greater than 1 µm in and a width of 0.24–0.4 µm. The length of these 2D copper nanosheets was longer than that found when using NaOH as a reducing agent. However, the width of 2D copper nanosheets was formed randomly. In addition, aggregation of the small-sized 2D copper nanosheets that are difficult to measure in length and width was seen, as shown in Figure 3. However, 2D copper nanosheets were synthesized without the formation of copper nanoparticles as by-products, unlike when using a NaOH reducing agent. This is because LiOH is basically a weaker reducing agent than NaOH and could cause a slower reduction process. Therefore, LiOH, as a reducing agent, can start to react with copper precursor without the formation of unnecessary product. However, the use of LiOH is likely to control the concentration of DMSO_2_ simultaneously in order to reduce the difference in the width of 2D copper nanosheets.

**Figure 4:**
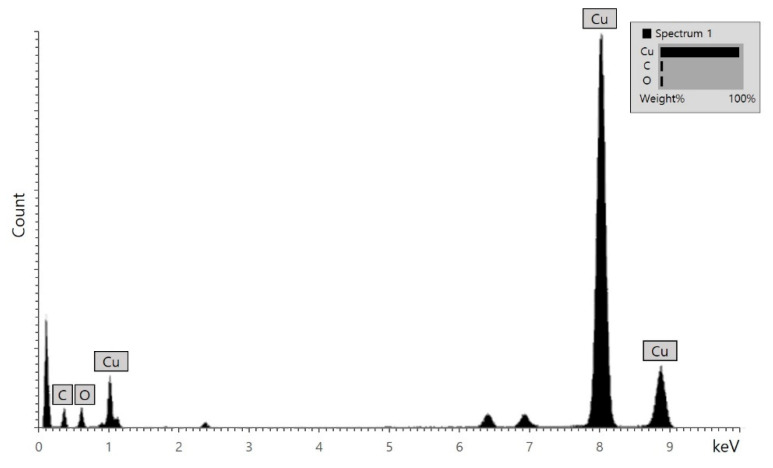
EDX spectrum of 2D copper nanosheets.

Figure 4 shows the energy-dispersive X-ray spectroscopy (EDX) pattern of 2D copper nanosheets. The element analysis from the EDX spectrum was conducted for copper (Cu) in 2D copper nanosheets. Some unexpected elements such as carbon (C) and oxygen (O) were revealed in the EDX spectrum, but with weak peaks. C detected from the TEM sample grid likely consisted of lacey carbon. O could be detected through the sealed thin window in the X-ray detector. It does not allow high counts, as shown in Figure 4. C and O peaks are not important for element analysis because the EDX spectrum results in the proportional weight percent. In Figure 4, a strong peak of Cu is observed at 8.04 keV, which means that the element of Cu is dominant in 2D copper nanosheets. Therefore, the formation of 2D copper nanosheets is confirmed by the presence of dominant Cu.

### 3.2. FTIR Study

**Figure 5:**
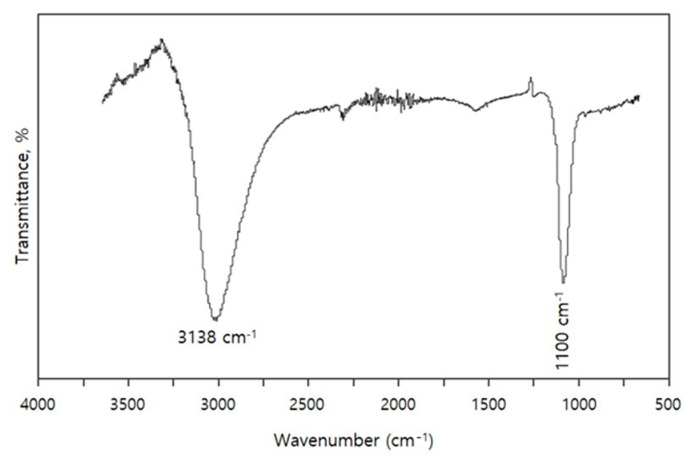
FTIR spectrum of 2D copper nanosheets synthesized with LiOH.

Figure 5 displays the FTIR spectrum of 2D copper nanosheets produced with LiOH. The region below 700 cm^−1^ has very small peaks that represent the vibration of Cu–O stretching and bending. The peak around 1100 cm^−1^ may reveal O–H bending vibration that is from either copper hydroxide (Cu(OH)_2_) generated during the copper ion reduction process or a LiOH reducing agent [28]. Therefore, 2D copper nanosheets could be reduced by a LiOH reducing agent. The strong and sharp peak at 3138 cm^−1^ indicates C–H bonding that is likely to be from the methyl group of DMSO_2_. Therefore, the successful use of DMSO_2_ to synthesize 2D copper nanosheets was confirmed by the observation of C–H bonding. The FTIR spectrum in Figure 5 indicates the presence of some amount of water bound to the 2D copper nanosheet solution [11,20].

### 3.3. UV–Vis Study

**Figure 6:**
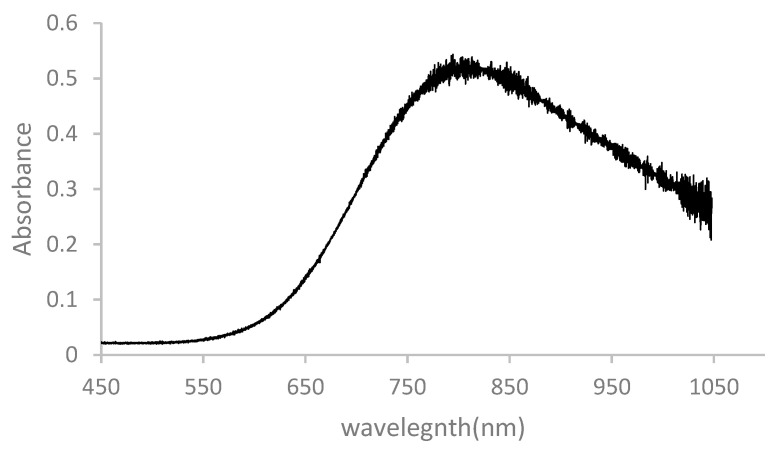
UV–Vis spectrum of 2D copper nanosheets synthesized with LiOH.

Figure 6 shows the UV–Vis spectrum of 2D copper nanosheets prepared by adding LiOH as a reducing agent. As shown in Figure 6, the change in absorbance to a longer wavelength, i.e., the redshift, indicates that elongated 2D copper nanosheets were produced [21]. Besides, the large aspect ratio of 2D copper nanosheets has a large peak shift. A better absorbance ability indicates a larger aspect ratio. Therefore, 2D copper nanosheets produced are likely to have a large aspect ratio and high absorbance ability. As shown in Figure 6, the absorption peak of 2D copper nanosheets was around 800 nm. 2D copper nanosheets can likely be used in laser photonic applications with a wavelength of 800 nm [29]. 

## 4. Conclusions

In conclusion, a new green synthesis method of 2D copper nanosheets was successfully developed by using methylsulfonylmethane (DMSO_2_), the use of which has not been reported before for the synthesis of nanomaterials. 2D copper nanosheets were formed in different morphologies with or without NaOH as a reducing agent and DMSO_2_ as a surfactant. With both NaOH and DMSO_2_, 2D copper nanosheets of 0.88 µm in length and 0.32 µm in width were obtained without aggregation. The concentration of DMSO_2_ was crucial for the formation of 2D copper nanosheets of large surface areas with regular edges. 2 M of DMSO_2_ was desirable for 2D copper nanosheets, resulting in 0.955 µm length and 0.42 µm width. The addition of LiOH instead of NaOH as a weak reducing agent and DMSO_2_ formed 2D copper nanosheets of 1 µm in length and 0.24–0.4 µm in width. Therefore, 2D copper nanosheets could be produced with the addition of either NaOH or LiOH as a reducing agent and DMSO_2_, which is an essential material. The EDX spectrum exhibited the presence of the dominant copper (Cu) in 2D copper nanosheets, as indicated by a strong peak of Cu. The FTIR study confirmed the formation of 2D copper nanosheets with DMSO_2_ by indicating C–H bonding. In addition, the UV–Vis spectrum of 2D copper nanosheets revealed a large peak shift and high absorbance ability. The light absorption peak at 800 nm indicates that this material has potential applications in laser photonics.

This study was focused on developing a new green synthesis method of 2D copper nanosheets. For future work, structural studies should be conducted, the material properties of 2D copper nanosheets should be evaluated, and the production of 2D copper nanosheets should be realized on a large scale for practical applications.

## Data Availability

The data presented in this study are available on request from the corresponding author.
